# Reframing Telomere Biology in Exercise Science: From Descriptive Metrics to Redox–Metabolic Mechanisms for Precision Healthy Aging (2000–2025)

**DOI:** 10.3390/biomedicines14061396

**Published:** 2026-06-21

**Authors:** Kun-Ho Lee, Kwon-Jae Song, Yun-A Shin

**Affiliations:** 1 Department of Sports Rehabilitation, Doowon Technical University, 159, Jurawi-gil, Paju-eup, Paju-si 10838, Republic of Korea; ksjudo1@doowon.ac.kr; 2 Epic Care Laboratory, Epic Care Research Institute, 62, Saemal-ro, Songpa-gu, Seoul 05841, Republic of Korea; 3 Department of Prescription and Rehabilitation of Exercise, Dankook University, 119, Dandae-ro, Dongnam-gu, Cheonan-si 31116, Republic of Korea

**Keywords:** telomere, exercise, oxidative stress, healthy aging, bibliometric analysis, epigenetic clock, precision medicine, HIIT

## Abstract

**Background/Objectives**: Telomeres are critical biomarkers of biological aging, with shortened leukocyte telomere length strongly linked to all-cause mortality and age-related disease risk. Although exercise modulates telomere dynamics, the field’s evolution from descriptive measurements to mechanistic inquiries involving redox biology and epigenetics remains incompletely mapped. This study systematically characterized the global research landscape of telomere–exercise science over 25 years to establish a strategic evidence base for precision exercise prescription. **Methods**: A bibliometric analysis was conducted on 858 publications from the Web of Science Core Collection (2000–2025). CiteSpace and VOSviewer were used for keyword co-occurrence analysis, strategic thematic mapping, and citation burst detection to visualize global research trends and identify emerging frontiers. **Results**: Annual publication volume grew from 2 (2000) to 71 (2025), with a compound annual growth rate of 15.4%. China emerged as one of the leading global contributors. Thematic analysis revealed a paradigm shift from descriptive leukocyte telomere length studies toward mechanistic investigations of oxidative stress, mitochondrial homeostasis, and epigenetic clocks. Keyword network analysis confirmed oxidative stress and inflammation as central hubs, mediating telomere protection via redox regulation and non-canonical telomerase functions. **Conclusions**: Exercise preserves telomere integrity primarily through redox–mitochondrial homeostasis, hormesis-driven antioxidant upregulation, and non-canonical telomerase activation. For aging populations and individuals at metabolic risk, aerobic training and high-intensity interval training (HIIT) are recommended as first-line non-pharmacological interventions for healthspan extension. Leukocyte telomere length and telomerase activity should be integrated as biomarkers in preventive medicine practice. Future large-scale randomized controlled trials incorporating multi-omics approaches and sex-stratified analyses are warranted to establish individualized dose–response guidelines for precision exercise prescription.

## 1. Introduction

Telomeres, the protective nucleoprotein structures composed of TTAGGG tandem repeats at the ends of eukaryotic chromosomes, play a pivotal role in maintaining genomic stability and regulating cell division. Due to the “end-replication problem” encountered by DNA polymerases during cell division, telomeres undergo progressive attrition. Once they reach a critical length, cells enter a state of replicative senescence or trigger apoptotic pathways. Consequently, telomere length is regarded as a “mitotic clock” predicting biological age and serves as a key biomarker for assessing the risk of various age-related pathologies, including cancer, cardiovascular diseases, and neurodegenerative disorders [1,2,3,4]. Recent evidence further highlights that shortened leukocyte telomere length (LTL) is strongly associated with increased all-cause mortality, identifying it as an important determinant of healthspan [5]. In the context of rapidly aging societies, telomeres are thus gaining prominence not merely as molecular markers but as integrative indicators reflecting individual aging velocity and functional health, serving as essential metrics for validating lifestyle interventions aimed at extending healthspan.

Traditionally, telomerase has been recognized for its canonical role in maintaining telomere length and chromosomal stability [6]. However, accumulating evidence [7] suggests that telomerase, particularly its catalytic subunit telomerase reverse transcriptase (TERT), exerts diverse non-canonical functions, including the regulation of mitochondrial function, oxidative stress responses, inflammation, apoptosis, and cellular survival pathways. Furthermore, dysregulated telomerase activation is a hallmark of many cancers, contributing to cellular immortalization and tumor progression. These findings indicate that telomerase functions not merely as a telomere-maintenance enzyme but also as a key regulator of cellular aging, metabolic homeostasis, and disease processes.

Physical exercise represents a potent non-pharmacological strategy for modulating the aging process, offering diverse benefits ranging from the maintenance of physical function and prevention of chronic diseases to the mitigation of systemic stress. A multitude of studies have consistently reported that regular physical activity contributes to telomere length preservation through biological pathways such as inflammation reduction, oxidative stress regulation, and mitochondrial quality control [8,9,10,11,12]. Meta-analytic evidence suggests that physically active individuals, particularly athletes, possess significantly longer telomeres than sedentary controls (SMD = 0.54, 95% CI 0.18–0.90) [13]. Similarly, individuals with higher cardiorespiratory fitness (VO2max) demonstrate significantly greater telomere length compared to those with below-average fitness levels (SMD = 0.36, *p* = 0.002) [14]. Collectively, these findings indicate that exercise functions not merely as a means of improving physical fitness but as a potent biological intervention capable of directly attenuating cellular aging processes. This protective effect is often attributed to the mechanism of “hormesis,” wherein exercise-induced transient generation of reactive oxygen species (ROS) stimulates the upregulation of antioxidant defense systems and induces telomerase activity, thereby conferring long-term protection to telomeres [15].

However, the relationship between exercise and telomere dynamics cannot be explained by a simple linear causality. Telomere responses vary significantly depending on intervention variables such as exercise type, intensity, frequency, and duration, as well as individual genetic predispositions and metabolic profiles [16,17]. Notably, recent academic interest has expanded beyond simple LTL measurements to the realm of “Epigenetic Clocks,” investigating how lifestyle factors influence epigenetic modifications like DNA methylation. This shift suggests that telomere biology should be interpreted through the lens of a broader aging network linked to mitochondrial metabolism, inflammation, and systemic metabolic homeostasis, rather than as an isolated phenomenon confined to the cell nucleus [18]. In essence, telomeres should be viewed as a central nexus linking metabolic dysfunction, functional decline, and biological aging.

While several systematic reviews and meta-analyses have addressed the exercise–telomere relationship, most have remained microscopic in scope, focusing on specific exercise modalities, populations, or limited biological mechanisms. Although the exercise–telomere field has experienced exponential growth over the past two decades, much of this development has been driven by a limited number of influential research groups (e.g., UCSF). Despite the substantial accumulation of publications, little is known about how these studies collectively form the intellectual structure of the field or how research themes have evolved from simple telomere length measurements to broader concepts such as epigenetic aging. Without a clear understanding of the field’s knowledge structure and collaboration networks, researchers may face an increased risk of duplication and missed opportunities for identifying emerging research directions. Therefore, there is a compelling need to utilize advanced bibliometric tools, such as CiteSpace and VOSviewer, to systematically and quantitatively elucidate research trends and identify critical research gaps [18].

To bridge this gap, this study presents a comprehensive bibliometric analysis of the exercise–telomere literature published between 2000 and 2025. Specifically, this study aims to (1) analyze annual research productivity and growth trends; (2) identify collaboration networks among countries, institutions, and authors to pinpoint influential research groups; and (3) visualize the evolution of core research themes and emerging hotspots through keyword analysis. By objectively mapping the global research landscape, this study aims to provide foundational data essential for establishing evidence-based “Precision Exercise Prescriptions” targeted at promoting healthy aging and extending healthspan.

## 2. Materials and Methods

### 2.1. Data Source and Search Strategy

Data were retrieved from the Web of Science Core Collection (WoSCC), a premier global database widely recognized for its high-quality bibliographic data in the fields of sports science and biomedicine [19]. WoSCC was selected as the primary data source because it provides standardized bibliographic records, comprehensive citation indexing, and strong compatibility with major bibliometric analysis software, including CiteSpace (v6.4.R2), VOSviewer (v1.6.20), and Bibliometrix (R package). Furthermore, WoSCC is considered one of the most reliable databases for identifying influential publications, citation relationships, and knowledge structures, making it particularly suitable for large-scale bibliometric investigations.

To ensure comprehensive coverage of the literature, a topic-based search (TS) was conducted for the period from 1 January 2000 to 15 January 2026.

The search strategy combined key terms related to “Telomere” and “Exercise” using Boolean operators as follows:

TS = (“telomere” OR “telomerase” OR “leukocyte telomere length” OR “LTL”)

AND > TS = (“exercise” OR “physical activity” OR “training” OR “endurance” OR “aerobic” OR “resistance” OR “strength training” OR “HIIT” OR “fitness” OR “VO2max”)

**Figure 1:**
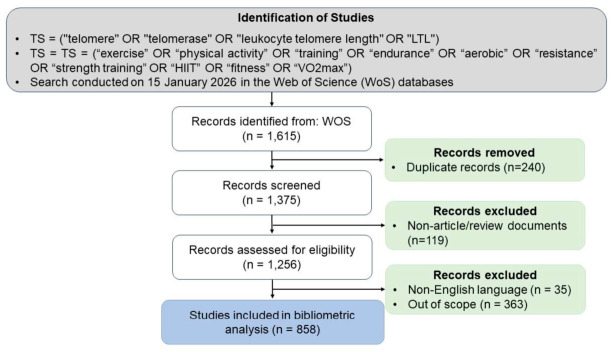
The flowchart of publication searching and screening.

To ensure the precision and academic rigor of the analysis, the search was restricted to original articles and review papers published in English. Other document types, such as meeting abstracts, editorials, letters, and proceeding papers, were excluded (Figure 1).

Although other databases, such as Scopus, PubMed, and Google Scholar, also contain relevant literature, variations in indexing policies, citation coverage, and metadata standardization across databases may affect data consistency and comparability in bibliometric analyses. Nevertheless, the exclusive use of WoSCC may have resulted in the omission of relevant studies indexed only in other databases, which should be considered when interpreting the findings.

### 2.2. Inclusion and Exclusion Criteria

Two independent researchers reviewed the titles and abstracts to select eligible literature based on the following criteria. Disagreements were resolved through discussion and consensus. Inclusion criteria were as follows: (a) studies focusing on telomere biology (e.g., length, telomerase activity) as a central theme; (b) studies investigating the effects of exercise, physical activity, training, or physical fitness as primary independent variables or interventions.; (c) studies involving human, animal, or cellular models, provided that exercise physiology remained the core analytical context; and (d) publications with complete bibliographic information (title, abstract, author, affiliation, journal, year). Exclusion criteria were (a) studies in which telomere measurements were not primary or secondary outcomes without substantive analysis; (b) general medical or molecular biology research lacking exercise or physical activity components; and (c) non-peer-reviewed articles or documents with incomplete or duplicate bibliographic data.

### 2.3. Data Extraction and Bibliometric Indicators

The retrieved data were exported in plain text format. Duplicates were removed using widely accepted bibliographic tools and manual inspection based on Digital Object Identifiers (DOIs). Extracted variables included publication year, authors, affiliations, countries, journals, citation counts, and keywords [20].

To evaluate research productivity and impact, we calculated standard bibliometric indicators, including total citations, average citations per year, H-index [21], and G-index. Additionally, structural metrics such as betweenness centrality and burst strength were analyzed to identify influential nodes within the network [22].

### 2.4. Bibliometric Analysis and Visualization Tools

This study employed a multi-tool approach to ensure a robust and multidimensional analysis. CiteSpace (version 6.4.R2) was used for document co-citation analysis and timeline visualization of research fronts. Specifically, the “Burst Detection” algorithm was applied to identify keywords and references that received a surge of attention during specific periods. VOSviewer (version 1.6.20) was used to visualize co-authorship networks (countries, institutions, authors) and keyword co-occurrence clusters. R Package “bibliometrix” (version 4.5.3) and Python (version 3.12) were used to generate strategic thematic maps based on density (development degree) and centrality (relevance degree). Themes were classified into four quadrants: motor themes, niche themes, emerging/declining themes, and basic themes, to evaluate the maturity and evolution of the field [23].

## 3. Results

### 3.1. Growth of Research Interest and Keyword Trends

From 2000 to 2025, a total of 858 publications regarding telomere and exercise were identified in the WoSCC database. The dataset comprised 679 original articles (79.1%) and 179 review papers (20.9%). The annual publication volume surged from 2 in 2000 to 71 in 2025, recording a Compound Annual Growth Rate (CAGR) of 15.4%, which reflects sustained and growing academic interest in this field.

**Figure 2:**
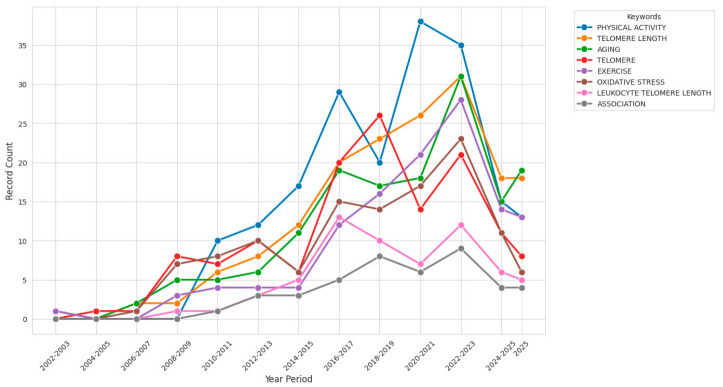
Annual trends in the frequency of the top eight core keywords in telomere–exercise research (2002–2025).

The temporal analysis of the top eight keywords reveals three distinct phases: incubation (2002–2009), growth (2010–2015), and rapid expansion (2016–2025) (Figure 2). These phases were identified based on annual publication growth and keyword frequency patterns. During the incubation phase, most core keywords appeared only sporadically and at low frequencies, reflecting the exploratory nature of early telomere–exercise research. The growth phase was characterized by a steady increase in the frequencies of key terms such as “physical activity”, “telomere length”, and “aging”, indicating expanding scientific interest and broader research participation. The rapid expansion phase was marked by accelerated publication growth and the emergence of mechanistic keywords, including “oxidative stress” and “leukocyte telomere length”, suggesting a transition from descriptive associations toward mechanistic and translational investigations.

### 3.2. Global Research Landscape

**Figure 3:**
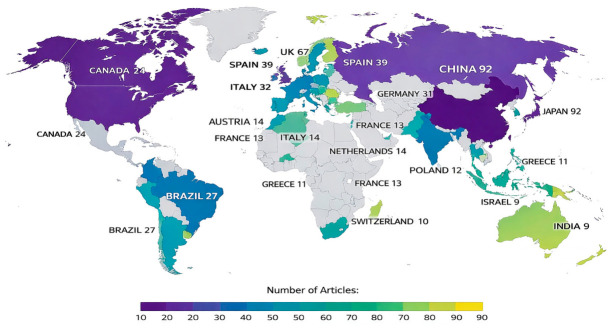
Top 20 most productive countries in telomere–exercise research.

An analysis of the top 20 most productive countries reveals a significant shift in the global research landscape (Figure 3). China emerged as the leading contributor, publishing 92 articles and securing the top rank globally, reflecting a significant contribution from Asian researchers. The United Kingdom followed with 67 articles, while Spain (39), Italy (32), and Germany (31) formed the upper tier of European contributions. The dominance of these top five nations highlights East Asia and Western Europe as the current hubs of exercise physiology and aging research. Notably, South Korea ranked 9th with 20 publications, marking it as the only other East Asian nation in the top 10 alongside China, demonstrating its significant regional contribution.

### 3.3. Intellectual Structure: Keyword Co-Occurrence Network

**Figure 4:**
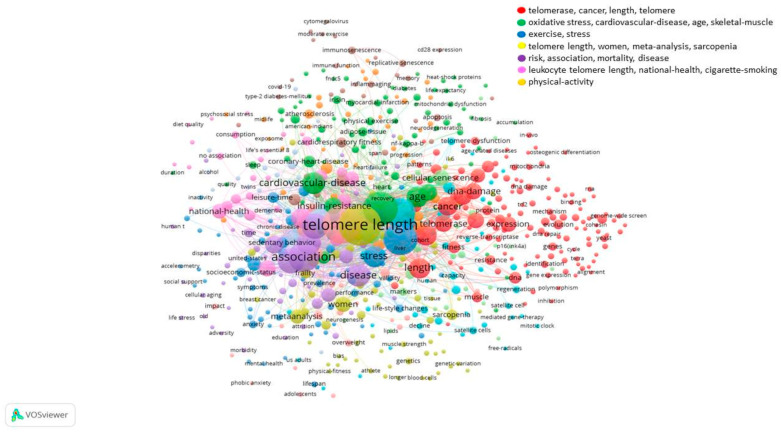
Keyword co-occurrence network of telomere-related publications (2000–2025).

The VOS viewer network analysis visualized the connectivity among core keywords, identifying seven major clusters that represent sub-themes of the field (Figure 4). The map identifies seven distinct clusters representing the intellectual substructure of the field. The central nodes—“Telomere length,” “Exercise,” and “Association”—act as bridges connecting diverse research topics. Red cluster focuses on molecular mechanisms and disease. This cluster investigates how exercise modulates telomerase activity to suppress tumorigenesis and repair DNA. Green cluster centers on oxidative stress and aging physiology. It highlights the physiological pathways where exercise mitigates cardiovascular risks and skeletal muscle aging by regulating redox status. Blue and apricot clusters relate to physical activity types and stress responses, linking exercise intensity to psychophysiological stress relief. Yellow represents specific populations and meta-analytic evidence. Purple and pink clusters focus on epidemiological associations and public health data, utilizing large-scale datasets to link lifestyle factors like smoking and obesity to telomere attrition.

### 3.4. Thematic Evolution and Maturation

**Figure 5:**
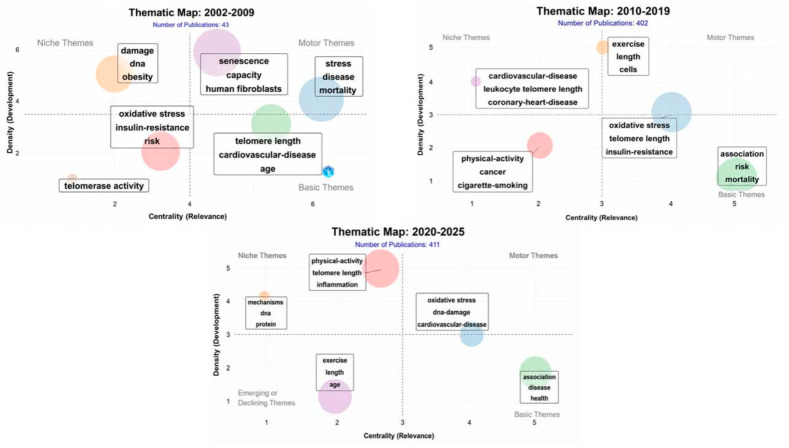
Strategic thematic maps illustrating the evolution of research themes across three time periods.

The strategic thematic map (Figure 5) illustrates the evolution of research themes across three time periods based on centrality (relevance) and density (development). During 2002–2009, research focused on basic biological mechanisms (“Senescence,” “Human fibroblasts”) in the Motor Themes quadrant, indicating an era driven by in vitro cellular studies. “Oxidative stress” appeared as a niche theme. During 2010–2019, the field expanded significantly. “Exercise” and “Telomere length” consolidated as motor themes. Crucially, “Oxidative stress” and “Insulin resistance” moved to basic themes, confirming that the metabolic regulation of telomeres by exercise became fundamental knowledge. During 2020–2025, the most recent period shows “Physical activity” and “Inflammation” dominating as motor themes. The shift from “Senescence” to “Physical Activity” indicates a transition from basic biology to clinical application. This confirms that the current research frontier prioritizes the preventive medicine perspective—how daily physical activity modulates chronic inflammation to delay aging. “Mechanisms” and “DNA” remain in the niche quadrant, pointing to highly specialized molecular inquiries.

### 3.5. Research Fronts and Citation Bursts

**Figure 6:**
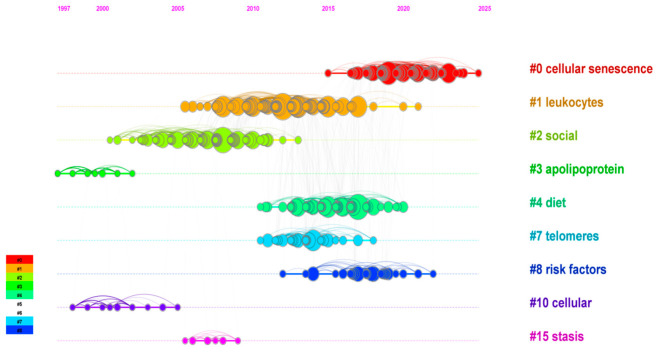
Timeline view of research fronts derived from CiteSpace.

The CiteSpace timeline view (Figure 6) visualizes the shifting research focus from socio-demographic factors to biological aging mechanisms. Early clusters like “#2 Social” and “#3 Apolipoprotein” faded after 2010. In contrast, “#0 Cellular Senescence” has formed a massive cluster of red nodes in the most recent period (2015–2025). The prominent “#0 Cellular Senescence” cluster in recent years indicates a major focus on cellular aging mechanisms. This indicates that the cutting edge of research has moved beyond simple length measurement to understanding how exercise delays the biological process of senescence itself. Furthermore, the analysis of top-cited references (Table 1) supports this trend. The most cited paper, “Epigenetic clock analysis of diet, exercise, education, and lifestyle factors [24]”, highlights the integration of lifestyle factors with epigenetic aging clocks, marking a paradigm shift in how biological age is quantified.

### 3.6. Influential Authors and Research Groups

The bibliometric assessment of the top 10 most influential authors within the exercise–telomere research domain reveals distinct patterns of productivity and scholarly impact, as summarized in Table 2. Among the prominent researchers identified, Lin, J. emerged as the leading figure, demonstrating superior performance across all evaluated metrics. Lin, J. ranked highest in productivity with 24 publications and achieved the most substantial citation impact, totaling 2227 citations. Furthermore, this author recorded the highest H-index (20) and G-index (24), while exhibiting the strongest collaborative network with a co-authorship total link strength of 208. Other significant contributors include Aviv, A., who attained the second-highest citation count (1717) despite a lower publication volume, and Kimura, M., who accrued 1347 citations. Institutional mapping indicates that high-impact research is primarily concentrated within three academic hubs: the University of California, San Francisco (Lin, J., Epel, E.S., and Blackburn, E.H.), the Harvard T.H. Chan School of Public Health (De Vivo, I. and Prescott, J.), and Rutgers New Jersey Medical School (Aviv, A. and Kimura, M.).

The analysis of high-impact literature in Table 1 further clarifies the field’s evolution regarding exercise, telomeres, and aging. The highest-cited work, “Epigenetic clock analysis of diet, exercise, education, and lifestyle factors” [24], has accrued 538 citations, underscoring the growing integration of lifestyle factors into biological aging research. Notably, the 2021 article “Physical activity, obesity and sedentary behavior in cancer etiology” [25] recorded the highest annual citation rate of 72.17, reflecting its immediate relevance to contemporary oncology. Thematic synthesis of these top-tier articles identified three primary research trajectories: the epidemiological associations between physical activity and leukocyte telomere length; molecular mechanisms involving oxidative stress, chronic inflammation, and cellular senescence; and the clinical application of epigenetic aging clocks and biological age biomarkers to evaluate longitudinal health outcomes.

## 4. Discussion

### 4.1. From Telomere Length to the Redox–Metabolic Axis

Our bibliometric analysis reveals a distinct structural evolution in telomere research: an initial focus on descriptive “telomere length” measurements has diversified into mechanistic clusters centered on “oxidative stress,” “metabolic risks” (insulin resistance/obesity), and “telomerase/TERT” before reintegrating into a holistic framework. The strong connectivity between oxidative stress and insulin resistance in the keyword network suggests that the field has transcended the simple “chromosome end replication” paradigm to embrace a macroscopic “redox–mitochondria–metabolic homeostasis” axis. This structural shift implies that the protective effects of exercise on telomere biology are not mediated by a single pathway but rather through a multidimensional network involving (1) long-term mitigation of oxidative stress and inflammatory burden; (2) reprogramming of mitochondrial function (biogenesis and ROS regulation); and (3) activation of both canonical and non-canonical (extra-telomeric) functions of telomerase/TERT [34,35,36,37].

### 4.2. Exercise Modalities and the Basis for Precision Prescription

The analysis of citation bursts and research fronts post-2015 indicates a transition from observing simple correlations between physical activity and telomere length to verifying modality-specific effects through randomized controlled trials (RCTs). The landmark 6-month RCT by Werner et al. [37] reported that endurance training and high-intensity interval training (HIIT) significantly increased telomerase activity (2- to 3-fold) and telomere length in peripheral blood mononuclear cells, whereas resistance training showed no such effects during the same intervention period. However, it is critical to interpret these findings within their methodological context.

Recent systematic reviews and meta-analyses provide a more comprehensive perspective on the exercise–telomere relationship. Overall, physically active individuals consistently exhibit longer telomeres and greater telomerase activity than sedentary counterparts, supporting a protective role of exercise in cellular aging [38,39]. Evidence from randomized controlled trials further suggests that aerobic-based exercise interventions, particularly endurance training and high-intensity interval training (HIIT), are associated with significant increases in telomerase activity and favorable effects on telomere maintenance [37,38,39]. In contrast, findings regarding resistance training remain inconsistent. A 2025 systematic review of RCTs reported significant improvements in telomerase activity following aerobic exercise (SMD = 0.33, *p* = 0.0001), whereas resistance exercise showed non-significant effects (SMD = 0.16, *p* = 0.43) [38]. However, this apparent discrepancy should be interpreted cautiously because resistance-training studies are relatively few in number and are often characterized by short intervention durations, small sample sizes, and methodological heterogeneity. Therefore, the current evidence is sufficient to support the beneficial role of aerobic exercise in telomere regulation but insufficient to conclusively determine whether resistance training exerts weaker biological effects or whether its benefits emerge through different mechanisms and longer-term adaptations [13,38,39].

These disparate outcomes suggest that aerobic-based exercise modalities (endurance and HIIT) offer more consistent telomere protection compared to resistance training in the short term. The underlying mechanisms likely involve differences in hemodynamic signaling: aerobic exercise generates sustained shear stress and nitric oxide (NO)-mediated anti-inflammatory signaling in the vascular endothelium, which may preferentially activate telomerase in circulating leukocytes [36,37,40].

In contrast, resistance training primarily induces mechanical tension and metabolic stress in skeletal muscle, which may influence telomere dynamics through different pathways or require longer durations to manifest measurable effects [38].

Nevertheless, resistance training remains essential for maintaining musculoskeletal function and metabolic health in aging populations. Future precision exercise prescriptions should prioritize aerobic and interval training for telomere-specific benefits while incorporating resistance training as a complementary modality to address sarcopenia and functional decline [36,38]. Critically, exercise duration of ≥16 weeks appears necessary for detectable effects on telomere biology, emphasizing the importance of sustained adherence [38,41].

### 4.3. Oxidative Stress and the Hormesis Hypothesis

The sustained high centrality of “oxidative stress” throughout the study period corroborates that telomere attrition is a dynamic physiological phenomenon sensitive to metabolic and oxidative environments, rather than merely a counter of cell divisions. Guanine-rich telomeric repeats are particularly vulnerable to oxidative DNA damage (8-OHdG), and chronic ROS overload accelerates senescence by triggering telomere fragility and repair failure [36,42]. However, exercise counters this via the mechanism of “hormesis.” While high-volume training without adequate recovery may exacerbate oxidative damage, regular exercise at appropriate intensities upregulates antioxidant enzymes (e.g., SOD, catalase) and enhances redox buffering capacity, thereby creating a long-term protective environment for telomeres [43,44]. This is consistent with our finding that “stress” and “dynamics” are classified as Basic Themes, confirming that the exercise–telomere relationship follows a strict dose–response characteristic [36].

However, the relationship between oxidative stress and telomere dynamics in vivo is more complex than previously assumed. A comprehensive 2023 meta-analysis of 37 studies encompassing 4969 individuals found that the overall correlation between oxidative stress markers and telomere dynamics was near zero (r = 0.027) [45]. This finding challenges the straightforward extrapolation from in vitro studies, where oxidative stress clearly accelerates telomere attrition [46,47].

Importantly, measurement methodology significantly influences this relationship. When analyses were restricted to studies using terminal restriction fragment (TRF) analysis—a more precise method than qPCR—a significant but modest correlation emerged (r = 0.09, *p* < 0.05) [45]. Furthermore, interventional studies manipulating oxidative stress showed a moderate effect on telomere dynamics (d = 0.36), supporting causality [45]. These findings suggest that oxidative stress does contribute to telomere attrition in living organisms, but the effect size is smaller and more context-dependent than in vitro models suggest [45,47].

The guanine-rich telomeric DNA remains particularly vulnerable to oxidative damage (8-oxo-dG formation), and chronic ROS overload can trigger telomere fragility and DNA damage responses [47,48]. However, the protective effects of exercise operate through hormetic mechanisms that extend beyond simple ROS reduction. Regular exercise at appropriate intensities upregulates endogenous antioxidant enzymes (SOD, catalase, and GPx) and enhances redox buffering capacity, creating a long-term protective environment [46,48]. Conversely, excessive training volume without adequate recovery may paradoxically increase oxidative damage, confirming the strict dose–response characteristic of the exercise–telomere relationship [45,46].

### 4.4. The Metabolic Loop and Non-Canonical Telomerase Functions

The recurrent emergence of “insulin resistance/obesity/diabetes” keywords highlights the formation of a bidirectional amplification loop between mitochondrial metabolic dysfunction and telomere impairment. This crosstalk operates through multiple interconnected pathways [34,49,50]:

Direction 1: Telomere dysfunction → Mitochondrial impairment. Critically short telomeres suppress the PGC-1α/β pathway, inhibiting mitochondrial biogenesis and reducing ATP production. Telomere-dysfunctional cells exhibit decreased mitochondrial mass, impaired oxidative phosphorylation, and increased dependence on glycolysis—a metabolic reprogramming that resembles the Warburg effect [34,51].

Direction 2: Mitochondrial dysfunction → Telomere attrition. Conversely, dysfunctional mitochondria generate excessive reactive oxygen species (ROS), which preferentially damage guanine-rich telomeric DNA and accelerate telomere shortening independent of cell division [34,49,52].

Mitochondrial dysfunction also induces epigenetic modifications (e.g., histone H4K8 acetylation) that further compromise telomere maintenance [52].

This mutual amplification creates a vicious cycle: telomere attrition impairs mitochondrial function, which in turn accelerates further telomere damage through oxidative stress [49,51,53]. Importantly, this cycle is not merely correlative but causally linked, as demonstrated by studies showing that primary mitochondrial dysfunction directly causes telomere shortening [51,52].

Exercise as a circuit breaker: Aerobic and interval training serve as potent interventions to break this vicious cycle by simultaneously (1) activating AMPK–PGC-1α signaling to enhance mitochondrial biogenesis and oxidative capacity; (2) reducing mitochondrial ROS production through improved electron transport chain efficiency; and (3) upregulating telomerase activity to counteract telomere attrition [34,37,38,39,46,49]. This multi-target mechanism explains why exercise effects on telomere biology cannot be attributed to a single pathway but reflect systemic metabolic reprogramming [34,53].

Furthermore, the catalytic subunit TERT exhibits non-canonical mitochondrial functions beyond nuclear telomere elongation. Under oxidative stress, TERT translocates to mitochondria, where it protects mitochondrial DNA (mtDNA), inhibits apoptosis, and enhances mitochondrial function [49,50]. RCT findings that endurance and interval training increase telomerase activity therefore demonstrate that exercise enhances cellular resilience itself, not merely telomere length maintenance [37,38]. This dual nuclear–mitochondrial function of TERT positions it as a central integrator of the exercise-induced anti-aging response [49,50].

### 4.5. Sex Differences in Exercise–Telomere Responses

Emerging evidence suggests that sex may modulate the relationship between physical activity and telomere dynamics, although findings remain preliminary. A 2025 meta-analysis of RCTs found a trend toward greater telomere length maintenance in females (SMD = 0.48, *p* = 0.06) compared to males (SMD = 0.38, *p* = 0.40), though neither reached statistical significance individually [38].

This pattern aligns with observational data showing that women generally have longer baseline telomeres than men, potentially due to estrogen-mediated telomerase activation [48].

Cross-sectional studies reveal complex sex-specific associations. In a large NHANES analysis, the protective association between vigorous leisure-time physical activity and longer telomere length was consistent across both sexes, but the magnitude of effect varied [54,55]. Notably, heavy smoking showed stronger associations with telomere shortening in women compared to men (P-interaction = 0.03), suggesting that women may be more susceptible to oxidative damage from certain exposures but potentially more responsive to protective interventions like exercise [56].

However, a 2025 systematic review focusing exclusively on women found inconsistent relationships between physical activity and telomere length, with positive associations observed only for specific modalities (combined aerobic and strength training, HIIT) and in specific age groups (early and late adulthood, but not middle adulthood) [57]. This age-dependent pattern may reflect hormonal transitions during perimenopause and menopause, which could modify telomere dynamics independently of physical activity [57].

Implications for precision prescription: These findings underscore the need for sex-stratified analyses in future RCTs and suggest that optimal exercise prescriptions for telomere protection may differ between men and women. The potential for greater responsiveness in women, if confirmed, could inform targeted interventions for female populations at risk for accelerated aging [38,56,57]. However, current evidence remains insufficient to justify sex-specific recommendations, highlighting this as a critical research gap.

### 4.6. Implications for Future Research and Practice

Synthesizing these bibliometric findings, we propose the following strategic directions for exercise prescription and future research:

First, telomeres should be utilized as integrative biomarkers reflecting metabolic–mitochondrial health, likely possessing high sensitivity for monitoring metabolic risk groups and aging populations [34,43]. Second, to maximize cytoprotective effects via telomerase activation, endurance and HIIT-based aerobic stimuli should be prioritized in exercise prescriptions. While resistance training is essential for muscular function, it should be applied complementarily in the context of telomere regulation [37,41]. Third, future studies must move beyond isolated telomere length analysis to adopt a multi-biomarker approach that simultaneously assesses telomerase activity, shelterin complex proteins, and DNA damage markers (8-OHdG). This comprehensive strategy will provide the rigorous foundation needed to validate the efficacy of exercise interventions for healthy aging and healthspan extension [34,36,58].

Nevertheless, several limitations should be acknowledged. This bibliometric analysis was based exclusively on the Web of Science Core Collection (WoSCC), and relevant studies indexed only in Scopus, PubMed, or Google Scholar may not have been included.

## 5. Conclusions

This bibliometric analysis systematically mapped the intellectual structure and developmental trajectory of telomere–exercise research from 2000 to 2025. The findings demonstrate a clear evolution of the field from descriptive investigations of leukocyte telomere length (LTL) toward mechanistic and translational research centered on oxidative stress, inflammation, mitochondrial regulation, and biological aging. In particular, oxidative stress and inflammation emerged as key mechanistic hubs linking exercise to telomere preservation, supporting the concept that regular physical activity promotes healthy aging through redox and metabolic homeostasis. The analysis further revealed a growing integration of telomere biology with emerging aging biomarkers, including telomerase activity and epigenetic aging clocks, reflecting a shift toward precision approaches for monitoring and optimizing healthspan. These trends position telomeres not merely as biomarkers of aging but as integrative indicators of cellular and metabolic resilience.

From a practical perspective, the current evidence supports the importance of aerobic-based exercise, particularly endurance training and high-intensity interval training (HIIT), as promising strategies for promoting telomere maintenance and healthy aging. However, substantial heterogeneity in exercise protocols and telomere assessment methods remains a major challenge for evidence synthesis.

Future research should prioritize standardized methodologies, large-scale randomized controlled trials, and integrative multi-omics approaches incorporating telomerase activity, DNA damage markers, and epigenetic signatures. Collectively, these findings reinforce exercise as a powerful non-pharmacological intervention for preserving telomere integrity and provide a scientific foundation for the advancement of precision exercise medicine.

## Figures and Tables

**Table 1:** Top 10 most highly cited publications in the exercise–telomere–aging research field.

Rank	Publication	Journal	Year	Total Citations	Avg. Citations per Year	Ref.
1	Epigenetic clock analysis of diet, exercise, education, and lifestyle factors	*Aging*	2017	538	53.80	[24]
2	Physical activity, obesity and sedentary behavior in cancer etiology: Epidemiologic evidence and biologic mechanisms	*Molecular Oncology*	2021	433	72.17	[25]
3	The association between physical activity in leisure time and leukocyte telomere length	*Archives of Internal Medicine*	2008	430	22.63	[26]
4	The epigenetic clock is correlated with physical and cognitive fitness in the Lothian Birth Cohort 1936	*International Journal of Epidemiology*	2015	418	34.83	[27]
5	Expression of p16INK4a in peripheral blood T-cells is a biomarker of human aging	*Aging Cell*	2009	385	21.39	[28]
6	A systematic review of leukocyte telomere length and age in adults	*Ageing Research Reviews*	2013	382	27.29	[29]
7	Childhood adversity heightens the impact of later-life caregiving stress on telomere length and inflammation	*Psychosomatic Medicine*	2011	338	21.13	[30]
8	Frailty and the role of inflammation, immunosenescence and cellular ageing in the very old: Cross-sectional findings from the Newcastle 85+ Study	*Mechanisms of Ageing and Development*	2012	329	21.93	[31]
9	p38 signaling inhibits mTORC1-independent autophagy in senescent human CD8+ T cells	*Journal of Clinical Investigation*	2014	318	24.46	[32]
10	Can physical activity ameliorate immunosenescence and thereby reduce age-related multi-morbidity?	*Nature Reviews Immunology*	2019	313	39.13	[33]

Avg.: average, Ref.: reference.

**Table 2:** Top 10 most influential authors in exercise–telomere research field.

Rank	Author	Affiliation	No. of Publications	Total Citations	Co-Authorship TLS	H- Index	G- Index
1	Lin, J	University of California, San Francisco, USA	24	2227	208	20	24
2	De Vivo, I	Harvard T.H. Chan School of Public Health, USA	16	1038	132	12	16
3	Denham, J	Edith Cowan University, Australia	15	382	54	9	15
4	Epel, E. S.	University of California, San Francisco, USA	12	1193	98	12	12
5	Codd, V.	University of Leicester, UK	12	306	94	7	12
6	Aviv, A.	Rutgers New Jersey Medical School, USA	11	1717	106	11	11
7	Prescott, J.	Harvard T.H. Chan School of Public Health, USA	11	733	88	9	11
8	Kimura, M.	Rutgers New Jersey Medical School, USA	9	1347	80	9	9
9	Blackburn, E. H.	University of California, San Francisco (UCSF), USA	9	834	63	9	9
10	Marti, A.	University of Navarra, Spain	8	251	56	7	8

TLS: total link strength, H-index: Hirsch index, G-index: Egghe’s G-index.

## Data Availability

The data that support the findings of this study are available from the Web of Science Core Collection (subscription required). Data derived from the study are available from the corresponding author upon reasonable request.

## Abbreviations

8-OHdG	8-Hydroxy-2′-deoxyguanosine
AMPK	AMP-activated protein kinase
CAGR	Compound annual growth rate
DOI	Digital Object Identifier
HIIT	High-intensity interval training
LTL	Leukocyte telomere length
mtDNA	Mitochondrial DNA
NO	Nitric oxide
PGC-1α/β	Peroxisome proliferator-activated receptor gamma coactivator 1-alpha/beta
qPCR	Quantitative polymerase chain reaction
RCT	Randomized controlled trial
ROS	Reactive oxygen species
SOD	Superoxide dismutase
TERT	Telomerase reverse transcriptase
TLS	Total Link strength
TRF	Terminal restriction fragment
TS	Topic-based search
UCSF	University of California, San Francisco
WoSCC	Web of Science Core Collection

## References

[1] Willeit P., Willeit J., Mayr A., Weger S., Oberhollenzer F., Brandstätter A., Kronenberg F., Kiechl S. (2010). Telomere length and risk of incident cancer and cancer mortality. JAMA.

[2] Fasching C.L. (2018). Telomere length measurement as a clinical biomarker of aging and disease. Crit. Rev. Clin. Lab. Sci..

[3] Liu B., Sun Y., Xu G., Snetselaar L.G., Ludewig G., Wallace R.B., Bao W. (2019). Association between Body Iron Status and Leukocyte Telomere Length, a Biomarker of Biological Aging, in a Nationally Representative Sample of US Adults. J. Acad. Nutr. Diet..

[4] Kalson N.S., Brock T.M., Mangino M., Fabiane S.M., Mann D.A., Borthwick L.A., Deehan D.J., Williams F.M.K. (2018). Reduced telomere length is associated with fibrotic joint disease suggesting that impaired telomere repair contributes to joint fibrosis. PLoS ONE.

[5] Goglin S.E., Farzaneh-Far R., Epel E.S., Lin J., Blackburn E.H., Whooley M.A. (2016). Change in Leukocyte Telomere Length Predicts Mortality in Patients with Stable Coronary Heart Disease from the Heart and Soul Study. PLoS ONE.

[6] Blackburn E.H., Epel E.S., Lin J. (2015). Human telomere biology: A contributory and interactive factor in aging, disease risks, and protection. Science.

[7] Shay J.W., Wright W.E. (2019). Telomeres and telomerase: Three decades of progress. Nat. Rev. Genet..

[8] Caspersen C.J., Powell K.E., Christenson G.M. (1985). Physical activity, exercise, and physical fitness: Definitions and distinctions for health-related research. Public Health Rep..

[9] Werner C., Fürster T., Widmann T., Pöss J., Roggia C., Hanhoun M., Scharhag J., Büchner N., Meyer T., Kindermann W. (2009). Physical exercise prevents cellular senescence in circulating leukocytes and in the vessel wall. Circulation.

[10] Mundstock E., Zatti H., Louzada F.M., Oliveira S.G., Guma F.T., Paris M.M., Rueda A.B., Machado D.G., Stein R.T., Jones M.H. (2015). Effects of physical activity in telomere length: Systematic review and meta-analysis. Ageing Res. Rev..

[11] Mora J.C., Valencia W.M. (2018). Exercise and older adults. Clin. Geriatr. Med..

[12] Ballin M., Nordström P. (2021). Does exercise prevent major non-communicable diseases and premature mortality? A critical review based on results from randomized controlled trials. J. Intern. Med..

[13] Valente C., Andrade R., Alvarez L., Rebelo-Marques A., Stamatakis E., Espregueira-Mendes J. (2021). Effect of physical activity and exercise on telomere length: Systematic review with meta-analysis. J. Am. Geriatr. Soc..

[14] Ryall C., Denham J. (2025). A Systematic Review and Meta-analysis Highlights a Link Between Aerobic Fitness and Telomere Maintenance. J. Gerontol. Ser. A.

[15] Jacome Burbano M.S., Gilson E. (2021). The Power of Stress: The Telo-Hormesis Hypothesis. Cells.

[16] Schellnegger M., Lin A.C., Hammer N., Kamolz L.P. (2022). Physical Activity on Telomere Length as a Biomarker for Aging: A Systematic Review. Sports Med.—Open.

[17] Sánchez-González J.L., Sánchez-Rodríguez J.L., Varela-Rodríguez S., González-Sarmiento R., Rivera-Picón C., Juárez-Vela R., Tejada-Garrido C.I., Martín-Vallejo J., Navarro-López V. (2024). Effects of Physical Exercise on Telomere Length in Healthy Adults: Systematic Review, Meta-Analysis, and Meta-Regression. JMIR Public Health Surveill..

[18] Nakagawa S., Samarasinghe G., Haddaway N.R., Westgate M.J., O’Dea R.E., Noble D.W.A., Lagisz M. (2019). Research Weaving: Visualizing the Future of Research Synthesis. Trends Ecol. Evol..

[19] Wang L., Chen Y., Shen W., Fan X., Jia M., Fu G., Chi X., Liang X., Zhang Y. (2023). A Bibliometric Analysis of Cardioembolic Stroke From 2012 to 2022. Curr. Probl. Cardiol..

[20] van Eck N.J., Waltman L. (2010). Software survey: VOSviewer, a computer program for bibliometric mapping. Scientometrics.

[21] Hirsch J.E. (2007). Does the H index have predictive power?. Proc. Natl. Acad. Sci. USA.

[22] Newman M.E. (2004). Coauthorship networks and patterns of scientific collaboration. Proc. Natl. Acad. Sci. USA.

[23] Callon M., Courtial J.-P., Turner W.A., Bauin S. (1983). From translations to problematic networks: An introduction to co-word analysis. Soc. Social. Sci. Inf..

[24] Quach A., Levine M.E., Tanaka T., Lu A.T., Chen B.H., Ferrucci L., Ritz B., Bandinelli S., Neuhouser M.L., Beasley J.M. (2017). Epigenetic clock analysis of diet, exercise, education, and lifestyle factors. Aging.

[25] Friedenreich C.M., Ryder-Burbidge C., McNeil J. (2021). Physical activity, obesity and sedentary behavior in cancer etiology: Epidemiologic evidence and biologic mechanisms. Mol. Oncol..

[26] Cherkas L.F., Hunkin J.L., Kato B.S., Richards J.B., Gardner J.P., Surdulescu G.L., Kimura M., Lu X., Spector T.D., Aviv A. (2008). The association between physical activity in leisure time and leukocyte telomere length. Arch. Intern. Med..

[27] Marioni R.E., Shah S., McRae A.F., Ritchie S.J., Muniz-Terrera G., Harris S.E., Gibson J., Redmond P., Cox S.R., Pattie A. (2015). The epigenetic clock is correlated with physical and cognitive fitness in the Lothian Birth Cohort 1936. Int. J. Epidemiol..

[28] Liu Y., Sanoff H.K., Cho H., Burd C.E., Torrice C., Ibrahim J.G., Thomas N.E., Sharpless N.E. (2009). Expression of p16(INK4a) in peripheral blood T-cells is a biomarker of human aging. Aging Cell.

[29] Müezzinler A., Zaineddin A.K., Brenner H. (2013). A systematic review of leukocyte telomere length and age in adults. Ageing Res. Rev..

[30] Kiecolt-Glaser J.K., Gouin J.P., Weng N.P., Malarkey W.B., Beversdorf D.Q., Glaser R. (2011). Childhood adversity heightens the impact of later-life caregiving stress on telomere length and inflammation. Psychosom. Med..

[31] Collerton J., Martin-Ruiz C., Davies K., Hilkens C.M., Isaacs J., Kolenda C., Parker C., Dunn M., Catt M., Jagger C. (2012). Frailty and the role of inflammation, immunosenescence and cellular ageing in the very old: Cross-sectional findings from the Newcastle 85+ Study. Mech. Ageing Dev..

[32] Henson S.M., Lanna A., Riddell N.E., Franzese O., Macaulay R., Griffiths S.J., Puleston D.J., Watson A.S., Simon A.K., Tooze S.A. (2014). p38 signaling inhibits mTORC1-independent autophagy in senescent human CD8^+^ T cells. J. Clin. Investig..

[33] Duggal N.A., Niemiro G., Harridge S.D.R., Simpson R.J., Lord J.M. (2019). Can physical activity ameliorate immunosenescence and thereby reduce age-related multi-morbidity?. Nat. Rev. Immunol..

[34] Gao X., Yu X., Zhang C., Wang Y., Sun Y., Sun H., Zhang H., Shi Y., He X. (2022). Telomeres and Mitochondrial Metabolism: Implications for Cellular Senescence and Age-related Diseases. Stem Cell Rev. Rep..

[35] Denham J. (2023). Canonical and extra-telomeric functions of telomerase: Implications for healthy ageing conferred by endurance training. Aging Cell.

[36] Arsenis N.C., You T., Ogawa E.F., Tinsley G.M., Zuo L. (2017). Physical activity and telomere length: Impact of aging and potential mechanisms of action. Oncotarget.

[37] Werner C.M., Hecksteden A., Morsch A., Zundler J., Wegmann M., Kratzsch J., Thiery J., Hohl M., Bittenbring J.T., Neumann F. (2019). Differential effects of endurance, interval, and resistance training on telomerase activity and telomere length in a randomized, controlled study. Eur. Heart J..

[38] Sun L., Zhang T., Luo L., Yang Y., Wang C., Luo J. (2025). Exercise delays aging: Evidence from telomeres and telomerase—A systematic review and meta-analysis of randomized controlled trials. Front. Physiol..

[39] Denham J., Sellami M. (2021). Exercise training increases telomerase reverse transcriptase gene expression and telomerase activity: A systematic review and meta-analysis. Ageing Res. Rev..

[40] Semeraro M.D., Smith C., Kaiser M., Levinger I., Duque G., Gruber H.J., Herrmann M. (2020). Physical activity, a modulator of aging through effects on telomere biology. Aging.

[41] Ludlow A.T., Ludlow L.W., Roth S.M. (2013). Do telomeres adapt to physiological stress? Exploring the effect of exercise on telomere length and telomere-related proteins. Biomed. Res. Int..

[42] Aguiar S.S., Rosa T.S., Neves R.V.P., Leite P.L.A., Maciel L.A., Gutierrez S.D., Rosa E.C., Andrade R.V., Degens H., Korhonen M.T. (2022). Telomere Length, SIRT1, and Insulin in Male Master Athletes: The Path to Healthy Longevity?. Int. J. Sports Med..

[43] Sahin E., DePinho R.A. (2012). Axis of ageing: Telomeres, p53 and mitochondria. Nat. Rev. Mol. Cell Biol..

[44] Ludlow A.T., Roth S.M. (2011). Physical activity and telomere biology: Exploring the link with aging-related disease prevention. J. Aging Res..

[45] Armstrong E., Boonekamp J. (2023). Does oxidative stress shorten telomeres in vivo? A meta-analysis. Ageing Res. Rev..

[46] Erusalimsky J.D. (2020). Oxidative stress, telomeres and cellular senescence: What non-drug interventions might break the link?. Free Radic. Biol. Med..

[47] Opresko P.L., Sanford S.L., De Rosa M. (2025). Oxidative Stress and DNA Damage at Telomeres. Cold Spring Harb. Perspect. Biol..

[48] Sack M.N., Fyhrquist F.Y., Saijonmaa O.J., Fuster V., Kovacic J.C. (2017). Basic Biology of Oxidative Stress and the Cardiovascular System: Part 1 of a 3-Part Series. J. Am. Coll. Cardiol..

[49] Vaurs M., Dolu E.B., Decottignies A. (2024). Mitochondria and telomeres: Hand in glove. Biogerontology.

[50] Zheng Q., Huang J., Wang G. (2019). Mitochondria, Telomeres and Telomerase Subunits. Front. Cell Dev. Biol..

[51] Gonzales-Ebsen A.C., Gregersen N., Olsen R.K. (2017). Linking telomere loss and mitochondrial dysfunction in chronic disease. Front. Biosci. (Landmark Ed).

[52] Guha M., Srinivasan S., Johnson F.B., Ruthel G., Guja K., Garcia-Diaz M., Kaufman B.A., Glineburg M.R., Fang J., Nakagawa H. (2018). hnRNPA2 mediated acetylation reduces telomere length in response to mitochondrial dysfunction. PLoS ONE.

[53] Tippairote T., Hoonkaew P., Suksawang A., Tippairote P. (2026). Chronic stress and the mitochondria-telomere axis: Human evidence for a bioenergetic-debt model of early aging. Biogerontology.

[54] Ogawa E.F., Leveille S.G., Wright J.A., Shi L., Camhi S.M., You T. (2017). Physical Activity Domains/Recommendations and Leukocyte Telomere Length in U.S. Adults. Med. Sci. Sports Exerc..

[55] Tucker L.A. (2017). Physical activity and telomere length in U.S. men and women: An NHANES investigation. Prev. Med..

[56] Vyas C.M., Ogata S., Reynolds C.F., Mischoulon D., Chang G., Cook N.R., Manson J.E., Crous-Bou M., De Vivo I., Okereke O.I. (2021). Telomere length and its relationships with lifestyle and behavioural factors: Variations by sex and race/ethnicity. Age Ageing.

[57] Page J., Stephens C., Richard M., Lyons E., Baumler E., Verklan M.T., Lorenzo E. (2025). The relationship between physical activity and telomere length in women: A systematic review. Mech. Ageing Dev..

[58] Sahin E., Colla S., Liesa M., Moslehi J., Müller F.L., Guo M., Cooper M., Kotton D., Fabian A.J., Walkey C. (2011). Telomere dysfunction induces metabolic and mitochondrial compromise. Nature.

